# PKCθ utility in diagnosing c-KIT/DOG-1 double negative gastrointestinal stromal tumors

**DOI:** 10.18632/oncotarget.19116

**Published:** 2017-07-08

**Authors:** Attila Kövecsi, Ioan Jung, Zoltan Szentirmay, Tivadar Bara, Tivadar Bara, Daniel Popa, Simona Gurzu

**Affiliations:** ^1^ Department of Pathology, University of Medicine and Pharmacy, Tirgu Mures, Romania; ^2^ Department of Pathology, Clinical County Emergency Hospital, Tirgu Mures, Romania; ^3^ Department of Molecular Pathology, National Institute of Onology, Budapest, Hungary; ^4^ Department of Surgery, University of Medicine and Pharmacy, Tirgu Mures, Romania; ^5^ Department of Pathology, CCAMF-Research Center, Tirgu Mures, Romania

**Keywords:** c-theta, protein kinase, GIST, diagnosis, CD117, Pathology Section

## Abstract

**Background:**

The aim of this study was to evaluate the diagnosis value of an immunohistochemical (IHC) panel of three antibodies for the diagnosis of gastrointestinal stromal tumors (GISTs).

**Material and methods:**

In 80 consecutive GISTs without lymph node metastases, the IHC examinations were performed using the antibodies CD117 (c-KIT), DOG-1 and c-theta (PKCθ) protein. The diagnostic value of PKCθ in c-KIT/DOG-1 negative GISTs has been explored in fewer than 10 Medline-indexed papers.

**Results:**

The c-KIT, PKCθ and DOG-1 positivity was noted in 92.50% (*n* = 74), 90% (*n* = 72) and 76.25% (*n* = 61) of the cases, respectively. All of the C-KIT negative cases (*n* = 6) were also DOG-1 negative but displayed PKCθ positivity. All of the DOG-1 positive cases (*n* = 61) also expressed c-KIT. No correlation between the examined markers and clinicopathological parameters was noted.

**Conclusions:**

The PKCθ sensitivity is similar to c-KIT and superior to DOG-1 sensitivity. All of the c-KIT/DOG-1 negative GISTs seem to express PKCθ. For a proper diagnosis of GIST, the c-KIT/DOG-1/PKCθ panel should be used, with possible therapeutic but not prognostic value.

## INTRODUCTION

Gastrointestinal stromal tumor (GIST) is the most common mesenchymal tumor of the gastrointestinal tract, with a 10-15 per million per year global incidence [1]. It is more frequently diagnosed in the fifth to seventh decades with an approximately equal gender distribution. The most frequent location is the stomach (55-60%), followed by small intestine (30-35%), colorectal segments (4-6%) and esophagus ( < 1%); rarely, GISTs may develop in the mesentery or retroperitoneum, when they are classified as extra-gastrointestinal GISTs (E-GIST) [2, 3].

Morphologically, GISTs may display several types of architecture. The most frequent type is the spindle cell architecture (70%), followed by epithelioid (20%) and mixed type (10%). In daily practice, the diagnosis of GIST is mainly based on the immunohistochemical (IHC) marker c-KIT (CD117). Due to its possible positivity in other tumors, such as melanomas, adenoid cystic carcinomas, Merkel cell carcinomas, Kaposi sarcomas, liposarcomas or leiomyomas/leiomyosarcomas, additional markers such DOG-1 (discovered on GIST-1) are used in most of the pathology laboratories [4, 5]. Those tumors that are negative for both of these markers, although they have *KIT* or *PDGFRA* mutations, are difficult to diagnose and remain unrecognized, although they could respond to Imatinib [6–10]. For this reason, new proteins are proposed to support the GIST diagnosis.

One of the relatively new markers described that are displayed by the c-KIT negative GISTs is the protein kinase C-θ (PKCθ), which is also known as c-theta protein. It is a serine-threonine protein kinase involved in T-cell activation and survival, skeletal muscle signal transduction and differentiation, nerve-muscle interaction, neuronal differentiation, cell proliferation, cancer cell-stroma interaction, transcription and apoptosis [6–10]. As the exact role of PKCθ in GIST is unknown, this IHC marker has not yet been approved for the daily diagnosis of GISTs.

The aim of the study was to analyze the diagnostic sensitivity of the c-KIT, DOG-1 and PKCθ expression in GIST and to perform a review of the 15 representative papers indexed in the Medline database (published between June 2004 and March 19, 2017) in the field of the supposed diagnostic value of the PKCθ [6–20]. Only six of these papers took into account the three markers [7, 9, 14–16, 18]. The other nine [6, 8, 10, 11–13, 17, 19, 20] were focused on correlation between c-KIT and PKCθ, without taking into account the DOG-1 expression.

## RESULTS

### Correlation between the IHC markers and clinicopathological factors

The median age of the patients ranged between 19 and 80 years (61.58±11.84 years). The other clinicopathological characteristics are shown in Table 1. All the cases had no lymph node metastasis and were negative for desmin. Distant metastases were identified in liver (*n* = 5) and peritoneum (*n* = 6).

**Table 1:** Clinicopathological characteristics of patients

Variable	*n*=80
***Age (years)***	61.58±11.84 (range 19-80 years)
***Gender***: Male/Female	35/45 (1:1.28)
***Tumor size*** (Median: 6.47±4.67 cm, range 0.4 to 21 cm)	
≥5 cm	45
<5cm	35
***Mitoses*** (50HPF) (Median: 8.43±14.02, range 0 to 89)	
≥5	29
<5	51
***Tumor location***	
Stomach	35
Small intestine	25
Colorectum	6
E-GIST	14
***Histological pattern***	
Spindle cell	64
Epithelioid cell	2
Mixed	14
***Risk group***	
Very low	10
Low	21
Intermediate	16
High	33
***Ki67 index***	
Low (≤5%)	60
High (>5%)	20
***Local invasion***	
present	14
absent	66
***Distant metastases***	
present	11
absent	69
***Necrosis***	
present	32
absent	48

The positive rates of c-KIT and DOG-1 in GISTs were 92.50% (74/80) and 76.25% (61/80) respectively. PKCθ positive staining was detected in 72/80 (90%) cases.

The expression of c-KIT and PKCθ has no significant correlation with clinicopathological parameters including gender, age, tumor size, mitotic rate, tumor location, histological type, risk degree, local invasion or presence of distant metastasis or intratumoral necrosis (Table 2).

**Table 2:** Correlation of the immunohistochemical expression of c-KIT, DOG-1 and PKCθ with the clinicopathological parameters (NA=non-available)

	*n*	c-KIT				DOG-1				PKC-theta			
		-	+	OR (CI:95%)	*p*	-	+	OR (CI:95%)	*p*	-	+	OR (CI:95%)	*p*
**Gender**													
Male	35	5	30	7.33 (0.81-66.00)	0.08	9	26	1.21 (0.43-3.40)	0.79	1	34	0.15 (0.01-1.36)	0.07
Female	45	1	44			10	35			7	38		
**Age (years)**													
≤45	8	0	8	0.60 (0.03-11.67)	0.98	2	6	1.07 (0.19-5.84)	0.98	0	8	0.44 (0.02-8.44)	1
>45	72	6	66			17	55			8	64		
**Tumor size**													
≥5 cm	45	4	41	1.61 (0.27-9.34)	0.69	11	34	1.09 (0.38-3.09)	0.95	5	40	1.33 (0.29-6.00)	1
<5cm	35	2	33			8	27			3	32		
**Mitotic rate (50HPF)**													
High (≥5)	29	2	27	0.87 (0.14-5.07)	0.95	5	24	0.55 (0.17-1.72)	0.41	2	27	0.55 (0.10-2.95)	0.49
Low (<5)	51	4	47			14	37			6	45		
**Tumor location**													
Stomach	35	2	33	NA	0.09	10	25	NA	0.02	3	32	NA	0.61
Small intestine	25	1	24			3	22			3	22		
Colorectum	6	2	4			4	2			1	5		
E-GIST	14	1	13			2	12			1	13		
**Histological pattern**													
Spindle cell type	64	4	60	NA	0.53	15	49	NA	0.66	6	58	NA	0.82
Epithelioid cell type	2	0	2			0	2			0	2		
Mixed type	14	2	12			4	10			2	12		
**Risk group**													
Very low	10	1	9	NA	0.80	3	7	NA	0.95	3	7	NA	0.14
Low	21	1	20			5	16			1	20		
Intermediate	16	2	14			4	12			1	15		
High	33	2	31			7	26			3	30		
**Ki67 index**													
Low	60	2	58	0.13 (0.02-0.82)	0.03	13	47	0.64 (0.20-2.01)	0.54	7	53	2.50 (0.28-21.75)	0.40
High	20	4	16			6	14			1	19		
**Local invasion**													
positive	14	2	12	2.58 (0.42-15.74)	0.28	2	12	0.48 (0.09-2.37)	0.49	1	13	0.64 (0.07-7.53)	0.69
negative	66	4	62			17	49			7	59		
**Distant metastasis**													
present	11	0	11	0.42 (0.02-8.06)	0.58	2	9	0.67 (0.13-3.45)	0.64	1	10	0.88 (0.09-7.98)	0.91
absent	69	6	63			17	52			7	62		
**Necrosis**													
present	32	3	29	1.55 (0.29-8.22)	0.67	8	24	1.12 (0.39-3.19)	0.95	2	30	0.46 (0.08-2.47)	0.37
absent	48	3	45			11	37			6	42		

Out of all the examined clinicopathological parameters, DOG-1 was only correlated with tumor location. Although the DOG-1 positive cases have predominated, significant positivity was noted for the GIST that involved the small intestine or retroperitoneal area (Table 2).

### Correlation between the four examined IHC markers

The value of the Ki67 index was directly correlated with c-KIT expression without correlation with DOG-1 or PKCθ (Table 2).

Out of the examined cases, 70% (56/80) expressed all the three examined markers: c-KIT, DOG-1 and PKCθ. All of the 61 DOG-1 positive cases and 13 of the 19 DOG-1 negative GISTs (68.42%) displayed c-KIT positivity (*p* = 0.0001).

**Figure 1:**
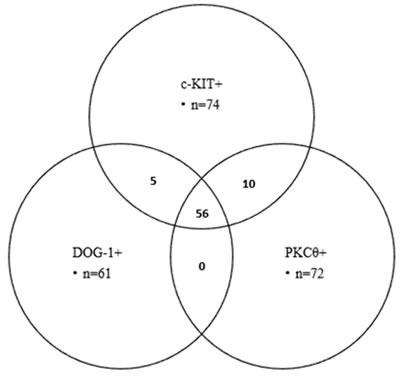
Correlation between c-KIT, DOG-1 and PKCθ expression revealed by Venn-diagram

PKCθ was expressed in 66 out of the 74 c-KIT positive GISTs and 56 out of the 61 DOG-1 positive cases (89.18% and 91.80% respectively). All of the six DOG-1 negative/ c-KIT negative cases expressed PKCθ. From the 13 DOG-1 negative/c-KIT positive cases, 10 cases (76.92%) displayed diffuse PKCθ positivity. All of the eight PKCθ negative GISTs were positive for c-KIT; five out of eight cases also expressed DOG-1 (Figure 1).

## DISCUSSION

In patients with GIST, the previously published papers showed a c-KIT positivity rate of 80-100%, in line with the present study [8, 19, 22, 23]. The c-KIT negative cases were reported to be more frequently located on the stomach (96% of all negative GISTs) and displaying epithelioid or spindle cell-type architecture [7, 19]. In the present study, regardless of the tumor's location, the two epithelioid-type GISTs were c-KIT negative.

DOG-1 is a transmembrane protein located on the 11q13 chromosome that was reported to be IHC-expressed in 57-96% of GISTs [9, 14, 23]. Its expression is directly correlated with c-KIT positivity [14]; all of the DOG-1 positive cases expressed c-KIT in our material but not all of the c-KIT positive GISTs were also positive for DOG-1. Usually, DOG-1 does not mark other tumors, such as leiomyomas/leiomyosarcomas, melanomas, schwannomas, malignant peripheral nerve sheath tumors, inflammatory fibroid polyps, small cell carcinomas, Merkel cell carcinomas or seminomas [7, 15, 24]. Uncommonly, DOG-1 sporadic positivity was reported for renal tubes, eccrine glands and hair follicles. Some tumors such as dermatofibrosarcomas, uterine-type retroperitoneal leiomyomas (8%), peritoneal leiomyomatoses (23%), leiomyosarcomas and other soft tissue tumors with histiocytic or lipomatous differentiation, carcinomas of the esophagus (60%), stomach (26%) and colorectal segments (5%), basal cell carcinomas (6%), squamous cell carcinomas (21%), hepatocellular carcinomas, adenoid cystic carcinomas, synovial sarcomas (16%) and desmoplastic melanomas (1%) also displayed DOG-1 positivity [9, 15, 23–26].

PKCθ was previously reported to be expressed in the interstitial cells of the Cajal lineage, Auerbach's plexus, T-cells, mast cells, endothelial cells, lymphoid organs, nervous system, skeletal muscle, and 72-100% of GISTs, without positivity for other c-KIT negative soft tissue tumors, desmoid tumors or carcinomas [6, 8–10, 14, 20]. It is important to mention that the c-KIT positive non-GIST tumors, such as small or large cell carcinomas, renal chromophobe cell carcinomas, thymic carcinomas or seminomas, did not display PKCθ positivity [6, 10]. However, the referenced studies [6, 10, 20] only included 26-48 GISTs and 1-10 cases from the non-sarcomatous tumors. Weak PKCθ expression was reported by other researchers in 25-33% of leiomyomas, 6-28% of leiomyosarcomas, 33% of malignant peripheral nerve sheath tumors/Ewing sarcomas, 10-57% of gastrointestinal schwannomas (especially in Verocay bodies), 15% of desmoid tumors, more than one-third of melanomas and 10% of adenoid cystic carcinomas [6, 7, 9, 15, 19, 20].

In our study, all of the three IHC markers were expressed in 70% of GISTs and the sensitivity of the polyclonal c-KIT and PKCθ was nearly identical: 92.50% versus 90%, similar to some of the literature data [9]. The sensitivity of both markers was superior to the K9 clone of DOG-1 (76.25%). Other authors proved a similar sensitivity of c-KIT and DOG-1 but admitted a slightly greater c-KIT positivity for the tumors localized on the colorectal segments [23, 27] and a relatively higher sensitivity of the clone SP31 versus the commercial K9 clone of DOG-1 that was used in the present study (95% versus 90-94%) [9, 15]. The heterogeneity of the studies, the small number of examined cases and the paucity of the used clones induce discrepant results, with even a higher DOG-1 or PKCθ sensitivity, compared with c-KIT, being proved [6, 10, 15, 27]. The differences can also be explained by the predominance of the DOG-1 positive cases in the tumors of the small intestine and E-GISTs, proved by the present material. In other studies, the gastrointestinal GISTs predominated [10]. Moreover, none of our cases showed lymph node metastases.

The PKCθ diagnostic value seems to be important for the c-KIT negative cases [7, 9]. A 70-100% PKCθ positivity was previously reported in c-KIT negative GISTs regardless of *KIT* or *PDGFRA* status [8, 19, 20]. PKCθ also marked the *PDGFRA* mutant GISTs (for exons 12 or 18) that are negative for c-KIT and even the cases with myxoid stroma [6, 19]. Overexpression of the *PKCθ* gene and PKCθ expression at the RNA level were also displayed in GIST samples but not in other c-KIT positive non-GIST soft tissue sarcomas or other tumors [10, 20].

Although a correlation between c-KIT and DOG-1 was proved by most of the studies, discrepant results were founded for c-KIT negative cases. The c-KIT negative GISTs are 36-100% DOG-1 positive [15], express either DOG-1 or PKCθ or show double positivity for DOG-1 and PKCθ [7, 9] but can also be negative for both DOG-1 and PKCθ (2/5 cases) [9]. The uncommon DOG-1 negativity was proved in only 3-4% of c-KIT negative GISTs; they are usually wild type *KIT/PDGFRA* mutant cases [9]. To our knowledge, the diagnostic value of PKCθ in double c-KIT/DOG-1 negative GISTs was shown in only three papers: 2/26 [7], 1/5 [9] and 1/1 c-KIT negative GISTs [16]. In all of these cases, PKCθ positivity was proved, as in this material, which comprised the largest reported case series of c-KIT/DOG-1 negative GISTs displaying PKCθ positivity (6/6 c-KIT negative GISTs selected from 80 GISTs). In one of the studies, the molecular examinations performed in c-KIT negative GISTs showed mutations in: *KIT* exon 11 for DOG-1 negative/PKCθ negative GISTs (two cases), *PDGFRA* exon 18 for DOG-1 negative/PKCθ positive (one case) or DOG-1 positive/PKCθ positive GISTs (one case), and *PDGFRA* exon 12 for DOG-1 positive/PKCθ positive GISTs (one case) [9]. The PKCθ negative GISTs showed *c-KIT* mutations in exon 11 regardless of the other IHC markers [19].

Similar *PKCθ* gene expression levels were proved when detected with a goat polyclonal or a mouse monoclonal antibody [20]. It may be mandatory to prove the PKCθ positivity to confirm the diagnosis of GIST, but its negativity is uninformative [20] because all of the PKCθ negative GISTs display c-KIT and CD34 positivity [6], with/without DOG-1 expression [9], similar to our data.

In the present study, no one of the three examined markers proved to be an indicator of prognosis. They proved to be valuable as diagnostic tools only. The main limitations of this study are the small number of cases and absence of cases with lymph node metastases.

As the possible role of PKCθ in protecting T-cells from apoptosis and promoting activation of the immune cells’ inflammation, as well as in promoting multidrug resistance (MDR), was previously proved [10, 28, 29], we conclude that PKCθ might be a novel therapeutic target for the immune therapy of GISTs or a potential indicator of resistance to Imatinib. In c-KIT/DOG-1 negative GISTs, the PKCθ expression should be checked for a complex differential diagnosis.

## MATERIALS AND METHODS

### Tissue samples

The present retrospective study included 80 formalin-fixed and paraffin-embedded tissue samples of primary GISTs. The surgically removed GIST specimens from consecutive cases were retrospectively collected from the Department of Pathology of the Clinical County Emergency Hospital of Tirgu-Mures, Romania, from 2003 to 2015. No neoadjuvant chemotherapy was given prior to resection. The approval of the Ethical Committee of the University of Medicine and Pharmacy of Tirgu-Mures, Romania, was obtained for retrospective evaluation of the cases.

The morphological diagnosis of GIST was confirmed by two pathologists, the histological pattern was identified and the mitotic index was calculated. The main prognostic factors, such as size, mitotic index and anatomical location, were analyzed based on the NIH's modified consensus classification [21].

### Immunohistochemical analysis

For IHC analyses, tissue microarray (TMA) blocks were performed, containing three representative areas of each GIST tissue (3 mm diameter core). The IHC stains were performed using the antibodies c-KIT (rabbit polyclonal, DAKO Glostrup, Denmark, dilution 1:500), DOG-1 (mouse monoclonal, clone K9, Novocastra, Newcastle, UK, dilution 1:50), Ki67 (MIB-1 clone, DAKO, dilution 1:500), and PKCθ (polyclonal, ABCAM, dilution 1:200) according to the instructions of the manufacturer. The developing was performed with DAB (diaminobenzidine) solution (Novocastra). For the negative controls, incubation was conducted with the omission of specific antibodies [30].

**Figure 2:**
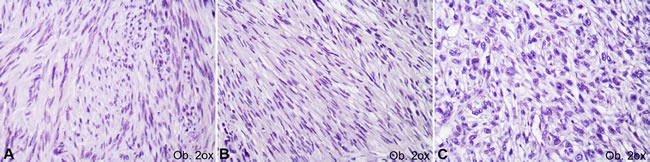
Negative immunoexpression of c-KIT (**A**), DOG-1 (**B**) and PKCθ (**C**) in gastrointestinal stromal tumors.

**Figure 3:**
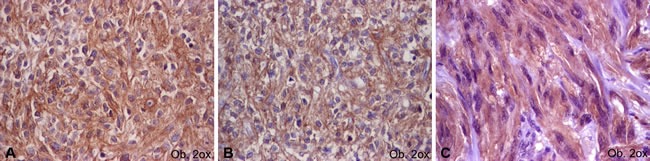
Positive immunoexpression of c-KIT (**A**), DOG-1 (**B**) and PKCθ (**C**) in gastrointestinal stromal tumors.

The cut-off value used was 5% for Ki67. The c-KIT, DOG-1 and PKCθ marked the cell cytoplasm with/without membrane positivity. In line with the previous studies, cases with positivity in a few single cells were considered negative (Figure 2); positive cases (Figure 3) showed focal or diffuse unequivocal positivity in several cell clusters or more than 10% of the tumor cells [7, 10]. The IHC assessment was performed independently by two pathologists.

### Statistical analysis

Statistical analysis was performed using the GraphPad InStat 3 software and two-sided tests. A *p*-value < 0.05 with 95% confidence interval was considered statistically significant.
